# Association of visual and quantitative heterogeneity of 18F-FDG PET images with treatment response in locally advanced rectal cancer: A feasibility study

**DOI:** 10.1371/journal.pone.0242597

**Published:** 2020-11-30

**Authors:** Paula Martin-Gonzalez, Estibaliz Gomez de Mariscal, M. Elena Martino, Pedro M. Gordaliza, Isabel Peligros, Jose Luis Carreras, Felipe A. Calvo, Javier Pascau, Manuel Desco, Arrate Muñoz-Barrutia

**Affiliations:** 1 Departamento de Bioingeniería e Ingeniería Aeroespacial, Universidad Carlos III de Madrid, Madrid, Spain; 2 Instituto de Investigación, Sanitaria Gregorio Marañón, Madrid, Spain; 3 Department of Pathology, Hospital General Universitario Gregorio Marañón, Madrid, Spain; 4 School of Medicine, Universidad Complutense, Madrid, Spain; 5 Department of Radiology and Medical Physics, Hospital General Universitario Gregorio Marañón, Madrid, Spain; 6 Department of Oncology, Hospital General Universitario Gregorio Marañón, Madrid, Spain; 7 Centro de Investigación Biomédica en Red de Salud Mental (CIBERSAM), Madrid, Spain; 8 Centro de Investigaciones Cardiovasculares Carlos III (CNIC), Madrid, Spain; Chang Gung Memorial Hospital at Linkou, TAIWAN

## Abstract

**Background and purpose:**

Few tools are available to predict tumor response to treatment. This retrospective study assesses visual and automatic heterogeneity from ^18^F-FDG PET images as predictors of response in locally advanced rectal cancer.

**Methods:**

This study included 37 LARC patients who underwent an ^18^F-FDG PET before their neoadjuvant therapy. One expert segmented the tumor from the PET images. Blinded to the patient´s outcome, two experts established by consensus a visual score for tumor heterogeneity. Metabolic and texture parameters were extracted from the tumor area. Multivariate binary logistic regression with cross-validation was used to estimate the clinical relevance of these features. Area under the ROC Curve (AUC) of each model was evaluated. Histopathological tumor regression grade was the ground-truth.

**Results:**

Standard metabolic parameters could discriminate 50.1% of responders (AUC = 0.685). Visual heterogeneity classification showed correct assessment of the response in 75.4% of the sample (AUC = 0.759). Automatic quantitative evaluation of heterogeneity achieved a similar predictive capacity (73.1%, AUC = 0.815).

**Conclusion:**

A response prediction model in LARC based on tumor heterogeneity (assessed either visually or with automatic texture measurement) shows that texture features may complement the information provided by the metabolic parameters and increase prediction accuracy.

## Introduction

Advances in disease diagnosis and treatment have improved the outcome of Locally Advanced Rectal Cancer (LARC). Nonetheless, most therapeutic decisions are still based on the Tumor, Node and Metastasis staging system (TNM), together with the distal and circumferential resection margin [1–6]. LARC tumors are a highly diverse group of lesions that may exhibit different responses to the same treatment, even in the same stage [7]. Therefore, early identification of responders to neoadjuvant treatment (NAT) could facilitate the development of tailored cancer therapies [4].

Medical imaging tools such as metabolic ^18^F-Fluorodeoxyglucose (FDG) PET imaging have become crucial in oncology for staging and treatment evaluation [8,9]. Over the past decades, ^18^F-FDG PET semi-quantitative metabolic activity descriptors derived from the Standardized Uptake Value (SUV), such as SUVmean and SUVpeak have been clinically used due to their prognostic ability [10–15]. More recent research recalls the interest of other parameters such as Total Lesion Glycolysis (TLG) and Metabolic Tumor Volume (MTV). These metrics provide information about metabolic activity in the whole volume. Indeed, TLG and MTV prognosis accuracy has been reported to be significantly higher than that of SUV values [16–18].

Nevertheless, the prognostic capacity of these metabolic features, even when combined with volume descriptors, is very limited. Over the last years, tumor heterogeneity has shown to be an additional source of information related with both prognosis and survival. It can be hypothesized that heterogenous phenotypes in the macroscopic scale can be related to underlying tumor pathophysiology and thus capture tumor aggressiveness [19].

Recently, radiomics has emerged as a way of quantifying tumor heterogeneity captured by radiological scans. The limited size of the available datasets for these purposes presents a limitation for deep learning approaches. Indeed, the approach of radiomics is still primarily based on handcrafted features used for regular machine learning predictive modelling [20]. Different radiomic based Texture Analysis (TA) approaches have attempted to objectively capture heterogeneity information from ^18^F-FDG PET imaging studies [21–26]. Several flaws affect the corroboration of texture analysis as a valuable predictor of therapeutic response, as most of the published studies up to date did not perform multivariate analysis of the texture analysis features nor performed a solid cross-validation [27]. Moreover, the quantitative texture features obtained are complicatedly related to both the pathophysiological tumor processes and the visual appearance of the images. This impairs a straightforward clinical usage of heterogeneity features.

The aim of this multi-disciplinary study was to retrospectively evaluate the predictive capacity of visually- and quantitatively assessed texture features in comparison with standard metabolic parameters. A visual assessment scale of tumor texture and an open-source and carefully revised workflow to automatize the texture analysis were introduced. Moreover, a multivariate analysis combined with cross-validation was applied to generate robust results and their clinical value was assessed.

## Materials and methods

### Patients

Thirty-seven LARC patients, either cT3-4 or cN+ according to the American Joint Committee on Cancer (AJCC), were selected. The inclusion criteria, staging and follow-up have been reported elsewhere [28]. Patients underwent an ^18^F-FDG PET/CT study before their treatment.

The study followed the recommendations of the Helsinki declaration and was approved by the Institutional Ethics Committee from Gregorio Marañon hospital. Signed informed consent from all patients was obtained and all images were anonymized.

### Treatment

All the patients followed the following treatment regime:

#### Neoadjuvant chemotherapy

Consisted in two FOLFOX cycles every two weeks. Each cycle consisted in Oxaliplatin 85mg/m^2^ on day one, intravenous Leucovorin 200mg/m^2^ on days one and two and intravenous 5-FU 400mg/m^2^ on days one and two.

#### Chemoradiotherapy

Two weeks after both cycles of chemotherapy, patients had five to six weeks of chemoradiotherapy (CRT). Pelvic radiotherapy was performed at a cumulative dose of 45–50.4 Gy (1.8 Gy daily fractions). Oral chemotherapy consisted in Tegafur at 1,200 mg/day on days one to four. Radiotherapy conformal three-dimensional plans followed the International Commission on Radiation Units and Measurements (ICRU) specifications and were delivered with 15 MV photon beams.

#### Surgery

Six weeks after CRT, resection was performed. Six senior surgeons participated. No strict criteria for surgical procedure was present but appropriateness of the safe distal margin distances and total mesorectal excision was mandatory.

#### Intraoperative radiotherapy

After surgery, patients received a 10–12.5 Gy intraoperative electron beam radiotherapy (IOERT) to the posterior pelvic cavity. Details have been already described elsewhere [28,29].

#### Postoperative chemotherapy

Adjuvant chemotherapy was selected consisting in either two FOLFOX cycles every two weeks or four to six cycles every four weeks of an intravenous 5- FU-370-425 mg/m^2^ and Leucovorin 20-25mg/m^2^/day in days one to five.

### Evaluation of treatment outcome

One pathologist examined all the resected specimens after NACT, CRT and surgery and evaluated the changes suffered after treatment following recommendations by Quirke *et al*. [30,31]. Specimens were staged according to the sixth edition of AJCC classification (ypTNM). The response to NAT was classified according to the tumor regression grade (TRG) scale [32]: TRG 0, no response; TRG 1, residual cancer outgrowing fibrosis; TRG 2, fibrosis outgrowing residual cancer cells; TRG 3, presence of residual cancer cells; and TRG 4, complete histopathological response. Applying this method, tumors were classified into NAT responders (TRG 3–4) or non-responders (TRG 0–2).

### PET/CT image acquisition protocol

Patients underwent PET/CT imaging before any of their treatments started. All the PET studies were obtained in the Nuclear Medicine department from Clinical La Luz de Madrid using a dedicated Philips Gemini TF model (standard bore, 70 cm) PET/CT simulator with an axial field of view = 18 cm (reconstructed field of view: 25,6,57.6 or 67,6 cm), and spatial resolution = 4.7 mm full-width half maximum. The scanner was equipped with a high light output scintillator (LYSO) that has high sensitivity, improved energy resolution and achieves a faster system timing resolution of approximately 600 ps, enabling better time of flight measurement.

3D PET/CT scans with 16-slice CT (slice thickness 3mm, reconstruction slice thickness 1mm and interval 1.5 mm) were acquired through the pelvis from the anal verge to the iliac crests in all patients. CT data was not acquired using a low-dose protocol. A rectal cancer CT scan protocol was used for volumetric analysis [33]. The contrast agent was not administered in these CT acquisitions. There are no differences in CT acquisitions among patients.

Whole-body PET emission images were acquired 45 min after intravenous injection of 5 MBq of FDG per kilogram of body weight. After radiotracer injection, patients rested and were orally hydrated (>0.5 L of water). Patient preparation included fasting for at least 6 h before the scan. In the morning of the scan day, patients were given a cleansing enema. PET data were normalized (to correct the system response) and corrected for attenuation, scatter radiation, random coincidences, dead time and decay. PET studies were normalized with respect to the blood glucose level measured before FDG administration [22,23]. Reconstruction was performed using weighted ordered subsets expectation maximization (2 iterations and 16 subsets) followed by the application of a smoothing filter (0.5 Hanning) and trilinear interpolation. The PET scans had a voxel size of 4x4x4 mm and a matrix size of 144x144x87 voxels.

### PET data analysis

**Fig 1:**
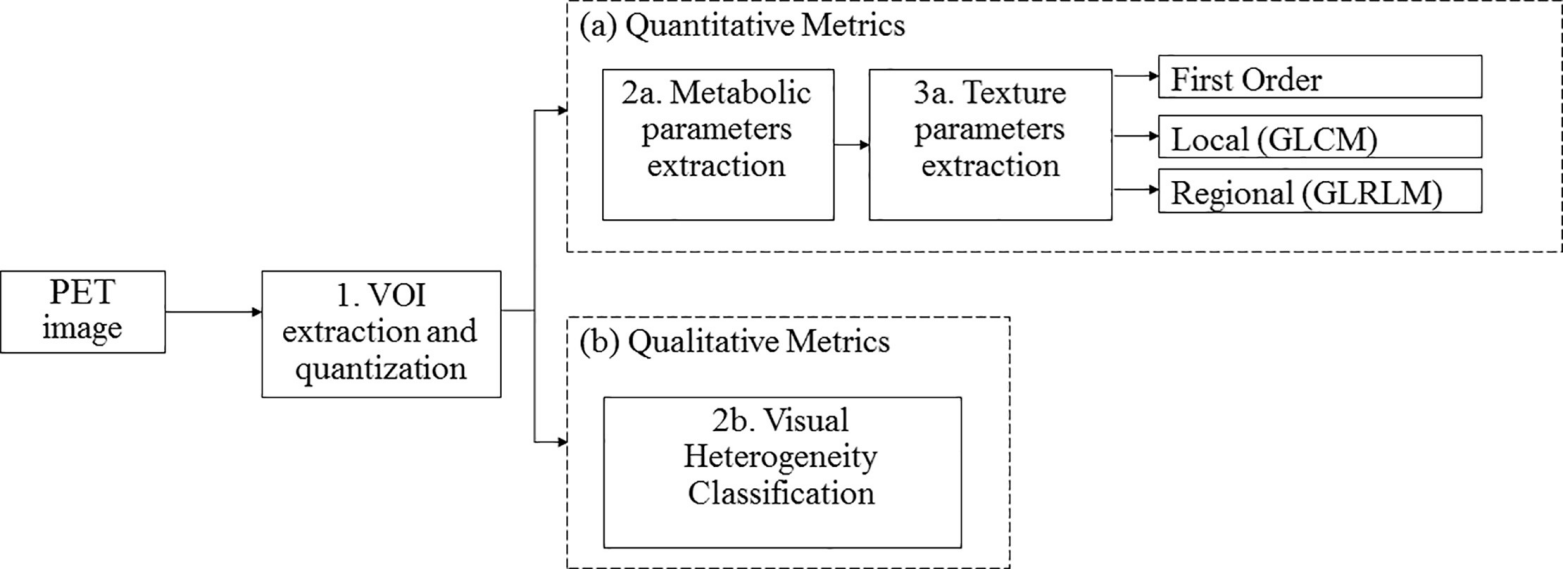
Summary of the workflow implemented for the estimation of the heterogeneity in the PET images: 1) The Volume of Interest (VOI) corresponding to the tumor is extracted and the image quantized to 64 levels; (a) Quantitative metrics are measured: 2a. The metabolic parameters described in Table 1; 3a. The texture features–First order, local (Gray Level Co-occurrence Matrix (GLCM)), regional (Gray Level Run Length Matrix (GLRLM)).

The processing workflow of this study is summarized in Fig 1. One experienced nuclear medicine specialist, blinded to the pathological status of the patients, obtained a Volume of Interest (VOI) by segmenting the tumor with a threshold of 40% of the maximum activity. Features related to tumor metabolism were calculated using 3-D Slicer open-source software Version 4.0.0. Harvard University, Cambridge, (MA) [34] and the PET-indiC module (Ethan Ulrich, University of Iowa). For verification, a second nuclear medicine specialist examined the VOIs based on the abovementioned scale and agreed on the classification performed.

Table 1 presents the complete list of metabolic parameters measured, classified into two sets: 1) standard clinical metabolic features (SUV_Max_, SUV_Peak_, Metabolic Tumor Volume (MTV) and Total Lesion Glycolysis (TLG)), referred to as CLINICAL, and 2) the complete set of metabolic variables, referred to as ALLMET. Characteristics referring to quartiles in glycolysis are the lesion glycolysis calculated from the respective quarter of the grayscale range of the tumour region.

**Table 1:** List of computed features from FDG-PET.

METABOLIC (ALLMET)				
CLINICAL:		REST:		
1. SUV_Peak_^a^ 2. SUV_Max_^a^ 3. Total Lesion Glycolysis (TLG)^a^ 4. Metabolic Tumor Volume (MTV)^a^			5. SUV_Mean_ 6. Standardized Added Metabolic Activity (SAM) 7. SUV_Min_ 8. First Quartile 9. Median 10. Third Quartile 11. Upper Adjacent	12. Q1 Distribution 13. Glycolysis Q1 14. Q2 Distribution 15. Glycolysis Q2 16. Q3 Distribution 17. Glycolysis Q3 18. Q4 Distribution Glycolysis Q4
**TEXTURE**				
GLOBAL:				
1. Maximum Intensity 2. Energy 3. Mean Intensity 4. Entropy 5. Median Intensity 6. Minimum Intensity 7. Range 8. Mean Deviation 9. Kurtosis			10. Root Mean Square 11. Variance 12. Standard 13. Deviation 14. Uniformity 15. Skewness 16Coefficient of Variation	
LOCAL (GLCM)^b^				
1. Energy 2. Entropy 3. Cluster Shade 4. Contrast 5. Cluster Prominence 6. Inverse Difference Momentum Normalized (IDMN)				
REGIONAL (GLRLM)				
1. Short-Run Emphasis (SRE) 2. Run-Length Non-Uniformity (RLN) 3. Long-Run Emphasis (LRE) 4. Run Percentage (RP) 5. Gray-Level Non-Uniformity (GLN) 6. Low Gray-Level Run Emphasis (LGLRE)	7. High Gray-Level Run Emphasis (HGLRE) 8. Long-Run Low Gray Level Emphasis (LRLGE) 9. Short-Run Low Gray Level Emphasis (SRLGE) 10. Long-Run High Gray Level Emphasis (LRHGE) 11. Short-Run High Gray Level Emphasis (SRHGE)			

Note: Q1-4 refers to the quartile.

^a^ denotes those parameters that belong to the CLINICAL set of metabolic features.

^b^ denotes the set of parameters that have been combined at five different offsets (odd distances from one to ten voxels).

### Heterogeneity visual assessment

**Fig 2:**
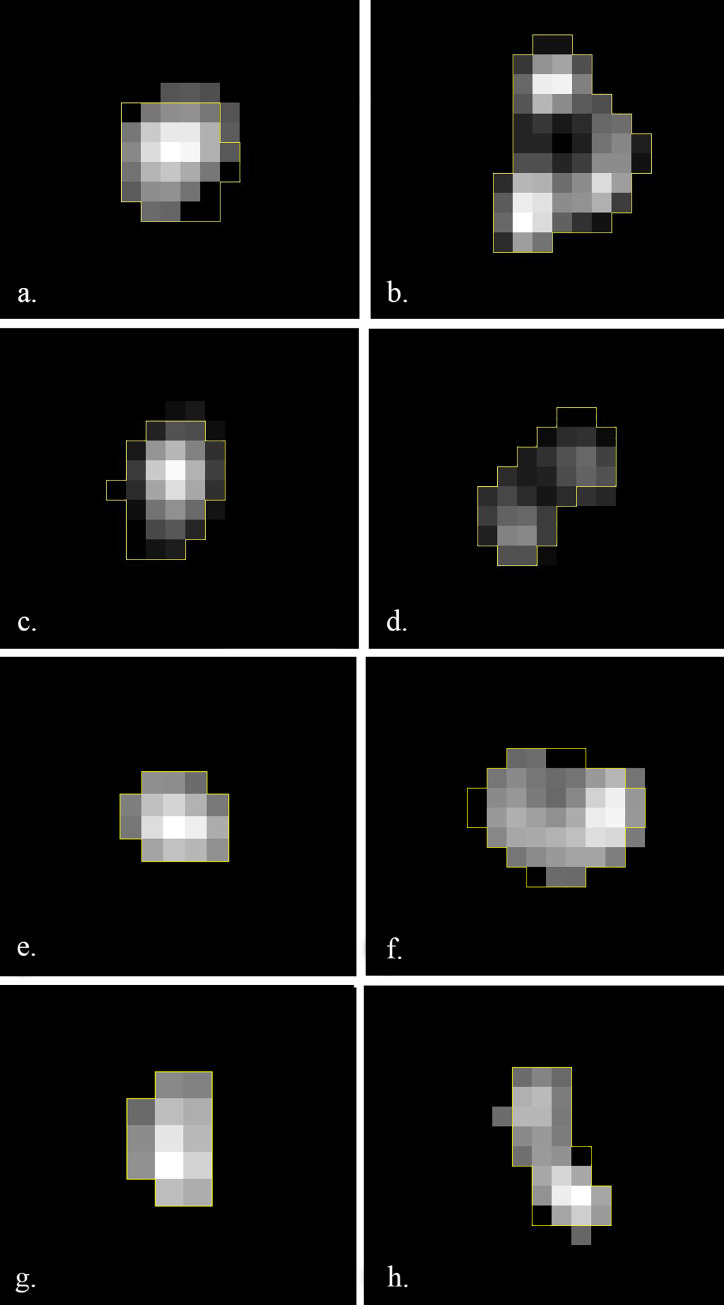
Major axial plane of the extracted VOI from four of the tumors analyzed with VOI boundaries shown in yellow. **(**a) and (c) show an example of homogeneous tumors with zero score in the visual heterogeneity scale; (b) and (d) show an example of heterogeneous tumors with score one in the visual heterogeneity scale. **(**e) and (g) show an example of tumors with zero score in the visual pattern scale; (f) and (h) show an example of tumors with score one in the visual pattern scale.

The same nuclear medicine specialist also classified all tumors in PET images according to two visual scales by examining the whole tumour volume (Fig 2). The ‘heterogeneity’ visual scale defined a zero (0) score when tumors were homogeneous in appearance or a one score (1) otherwise. The ‘pattern’ visual scale assigned a zero (0) score to nodular tumors and one (1) to multinodular or cavitated lesions. A second nuclear medicine expert examined the visual scoring and both found consensus on the final classification.

### Heterogeneity automatic assessment

Tumor VOIs were discretized to 64 gray levels as a way of normalizing the data, allowing interpatient comparison using the following equation [23]:

$$V(x)=64\frac{I(x)-\underset{i\in \Omega }{\min}\;i}{\underset{i\in \Omega }{max}\;i-\underset{i\in \Omega }{min}\;i+1}$$

where V is the intensity of the resampled image, I represents the intensity of the original image and Ω is the set of voxels inside the VOI. The range of 64 gray values has been previously identified as a tradeoff between noise removal and information loss [23]. Different image texture definitions were used to obtain the heterogeneity automatic analysis (Table 1). The complete set of automatic texture descriptors will be referred to as TEXTURE parameters.

In a first step, global texture features were extracted. They consist on a set of first and higher order statistics extracted from the gray-level histogram allowing the quantification of overall global changes in intensity within the VOI.

Secondly, intensity variations were studied with second-order or local texture features by using the gray level co-occurrence matrix (GLCM) [35]. Six statistics explaining local intensity variations were selected from the 21 originally described [35], based on previous literature to define the smallest set of GLCM features able to capture texture information [35–38]. To capture changes in local intensity beyond direct neighbors and reduce noise in the measurements [38,39], these features were calculated in a patch-wise manner using square kernels of different sizes, selected after examining the images and estimating the distance between voxels that characterized the texture pattern: 1,3,5,7 and 9 pixels.

Finally, intensity changes were studied using third-order or regional texture features using the Gray Level Run Length Matrix. Ten different statistics capturing regional texture measures were obtained from this matrix.

All texture features were calculated for the whole tumour volume. The first-order and third-order texture features were calculated using the Heterogeneity-CAD module (Narayan, V. *et al*, Harvard Medical School), from 3D Slicer [40,41]. We used in-house developed software to obtain GLCM texture metrics with respect to tumor volume, according to previous guidelines [35]. The Python software is available upon request.

### Immunohistochemistry staining and evaluation

A representative biopsy sample from each of the 37 patients was obtained for immunohistochemistry (IHC) procedures previous to the start of treatment. The standardization, preparation and staining was automatically done with a Dako Techmate device. Tumor sections were stained with commercially available monoclonal antibodies for key molecules in cancer. Some are involved in tumor growth, progression, proliferation, metastasis capacity and suppression (Namely, Vascular Endothelial Growth Factor Receptor-2 (VEGFR-2), Ki-67 protein, cyclooxygenase-2, E-cadherin and p-53 oncogene). Others are involved in cell apoptosis and growth (Namely, B-cell lymphoma-2 (BCL-2) and c-erb b2 oncogene).

The following preparations were used: VEGFR-2 (dilution 1:100, Flk-1; NeoMarkers), Ki-67 (prediluted MIB-1 clone; DAKO), COX-2 (dilution 1:200, clone RB9072-P; NeoMarkers), E-cadherin (prediluted; clone NHC38; DAKO), p-53 (dilution 1:50, IgG2b DO-7 clone; Novocastra), BCL2 (dilution 1:80, IgG1 bcl-2/100/D5 clone; Novocastra) and c-erb b-2 (dilution 1:40, IgG1 Clon 10A7; Novocastra). Slides were then evaluated in a light microscope at four representative areas at x20 and x40 magnification. Positive and negative controls were provided respectively by normal tissue and omission of antibody. The level expression of each marker in each patients’ sample was assessed by two pathologists in a quantitative percentage from 0 to 100 based on IHC staining presence and intensity. They were blinded to patients’ characteristics and to the rest of IHC biomarkers. Staining was re-evaluated later and the results were reproducible. The staining expression level was used for later comparison with radiomic features.

### Statistical analysis

Quantitative comparisons between responders and non-responders were carried out using the Mann-Whitney’s U test for continuous variables and χ^2^ test for discrete variables.

Correlation between features used for modelling is presented in S1 Fig. Stepwise multivariate binary logistic regression (Forward Wald’s, p< 0,05 for feature inclusion) was used to assess the predictive ability of parameters extracted from pretreatment 18F-FDG PET regarding patient´s response to NAT. To better validate and avoid over-fitting, multivariate binary logistic models were evaluated using a k-fold cross-validation (k = 5) where 80% of the dataset was used as training data and the remaining 20% was used as validation set. Mean accuracy and mean area under the ROC curve (AUC) from all the runs were used to assess the accuracy in the prediction of response, and 95% confidence intervals are reported in both cases.

The relationship between PET quantitative parameters and IHC biomarkers was assessed by means of Pearson correlation coefficient.

For evaluating the correlation between visual scales and automatically computed texture metrics, principal components were extracted from the automatically computed features for each patient scan. Contribution of each individual feature into the principal components can be found in S2 Fig. Principal components with eigenvalue greater than one were used. The correlation of the principal components with the visual scale was evaluated with the Spearman non-parametric correlation test.

## Results

### Baseline patient and tumour characteristics

**Fig 3:**
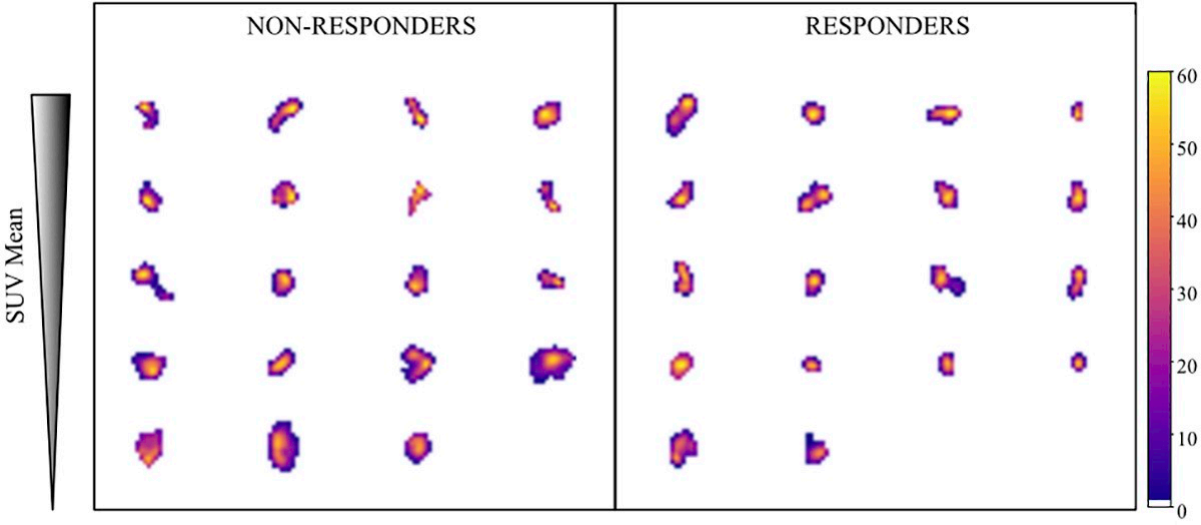
Comparison of the most representative slide for each patient from the resampled scans used for the heterogeneity analysis. Each slide was selected to contain the SUVmax in the tumour VOI of each patient. Non-responders and responders are located in the left and right sides while the vertical order is given by decreasing uptake–selected to be descending SUVmean.

No significant differences were found between treatment responders and non-responders in terms of clinical (age, gender, time between first PET scan and first NACT session, distance to anal verge and clinical staging risk group) and IHC characteristics (Table 2). Additionally, Fig 3 presents the most representative slice of each patient–selected to be the one containing SUV_max_ of the resampled scans used for the analysis.

**Table 2:** Comparison of the baseline clinical and immunohistochemistry (IHC) characteristics of the patients. The p-value corresponds to the χ^2^ test for gender and clinical staging risk group (degrees of freedom are 35 in both cases) and to the Mann-Whitney U test in the rest of variables.

	Variable	All patients (n = 37)	Responders (n = 18)	Non-responders (n = 19)	p—value
Clinical	Gender, n (%)				
	Male	26 (70.27%)	13 (72.22%)	13 (68.42%)	0.91
	Female	11 (29.73%)	5 (27.78%)	6 (31.58%)	
	Age (years), mean (standard deviation)	61.76 (8.65)	64.28 (7.63)	59.37(9.08)	0.07
	Time between scan and first NACT session (days), mean (standard deviation)	156.00 (40.67)	164.78 (45.96)	147.68(34.11)	0.13
	Distance to anal verge (cm), mean (standard deviation)	7.41 (3.29)	8.27 (3.95)	6.58(2.32)	0.12
	Clinical staging risk group, n (%)				
	Intermediate: T2 N1 or T3 N0	8 (21.62%)	5 (27,78%)	3 (15.79%)	0.66
	Moderately high: T3 N1, T4 N0	27 (72.97%)	12 (66.67%)	15 (78.95%)	
	High: T3 N2, T4 N1/2	2 (5.41%)	1 (5.56%)	1 (5.26%)	
IHC stains from diagnostic biopsy	Ki67, mean (standard deviation)	77.97 (17.62)	79.72 (16.13)	76.32 (19.21)	0.32
	p53, mean (standard deviation)	58.84 (40.97)	52.50 (40.59)	64.84(41.51)	0.15
	VEGFR, mean (standard deviation)	83.78 (13.44)	86.11 (28.73)	81.58(38.04)	0.43
	COX-2, mean (standard deviation)	58.78 (38.82)	51.94 (38.16)	65.26 (39.35)	0.12
	BCL—2, mean (standard deviation)	1.21 (6.60)	2.22 (9.43)	0.26 (1.15)	0.49
	CERB– 2, mean (standard deviation)	12.30 (26.60)	17.5 (35.82)	7.37 (12.29)	0.43
	E-cadherine, mean (standard deviation)	90.27 (13.64)	90.56 (14.34)	90.0(13.33)	0.41

### Visual scales for tumor response assessment

The comparison between responders and non-responders in the visual heterogeneity and pattern scales (Table 3) yielded statistically significant differences between groups (χ^2^ = 11.926, p = 0.003 in the case of visual heterogeneity and χ^2^ = 7,423, p = 0.013 in the case of visual pattern, degrees of freedom (dof) were 35 in both cases). The ‘heterogeneity’ visual scale is a dichotomic value that classifies the tumors in terms of their visual homogeneity in appearance. The ‘pattern’ visual scale classifies between nodular tumors and multinodular or cavitated lesions. They were correlated with r = 0.75 and p = 0.0012 (dof = 35).

**Table 3:** Comparison of the visual scoring system and PET parameters in responders and non-responders together with the χ^2^ test results and Mann-Whitney U test results respectively.

				Non-Responders			Responders			p- value (χ2)
Heterogeneity			Homogeneous (0)	5			14			0,003*
			Heterogeneous (1)	14			4			
Visual Pattern			Nodular (0)	2			9			0,013*
			Multinodular or cavitating(1)	17			9			
				Non- Responders			Responders			p- value (Mann-Whitney U)
				Median	Minimum	Maximum	Median	Minimum	Maximum	
Metabolic			SAM	112,554	44,545	367,764	77,916	13,093	270,784	0,049*
			TLG	112,554	44,545	367,764	77,916	13,093	270,784	0,049*
			Glycolysis Q1	42,948	11,036	164,781	27,009	5,426	96,47	0,042*
			Glycolysis Q2	38,388	9,487	127,259	24,796	3,883	120,262	0,061
			Q1 Distribution	45,337	21,594	62,6	44,929	36,301	57,692	0,641
Texture	Global	COV	0,227	0,203	0,356	0,245	0,2	0,534	0,013*
	Local (GLCM)	Distance one voxel	IDMN	0,459	0,328	0,582	0,4	0,309	0,59	0,039*
			Contrast	222,422	102,549	498,948	295,176	90,339	563,788	0,046*
		Distance three voxels	Energy	0,012	0,002	0,138	0,025	0,007	0,222	0,408
		Distance five voxels	Energy	0,111	0,006	0,262	0,048	0	0,175	0,036*
		Distance seven voxels	IDMN	0,179	0	0,525	0,116	0	0,172	0,035*
		Distance nine voxels	Cluster Shade	52,12	0	399,925	0,002	-38,334	323,58	0,050*
			Energy	0,012	0	0,171	0,015	0	0,108	0,766

Only those variables that remained significant (p-values marked with *) either here or in further analysis are shown. Note: SAM stands for Standardized Added Metabolic Activity, Q1 and Q2 refer to the first and second quartiles respectively, COV stands for Coefficient of Variation and IDMN stands for Inverse Difference Moment Normalize.

Accordingly, prognostic ability of the visual heterogeneity and pattern scales were statistically significant in the univariate binary logistic regression (*p* = 0.003 and *p* = 0.015, respectively). After cross validation, accuracy of prediction was 75,437±0,881% with an AUC of 0,759±0,009 for the visual heterogeneity scale and 69,268±0,890% with an AUC of 0,691±0,008 for the visual pattern scale.

When building a multivariate model to predict response including both visual scales, only visual heterogeneity remained statistically significant (*p* = 0.003). Furthermore, when the logistic regression model included together the heterogeneity visual scale and clinical metabolic features (CLINICAL), only the visual heterogeneity remained statistically significant (*p* = 0.003).

### Tumor response prediction using quantitative texture features

Responders and non-responders showed statistically significant differences for several metabolic and texture features (Table 2).

**Fig 4:**
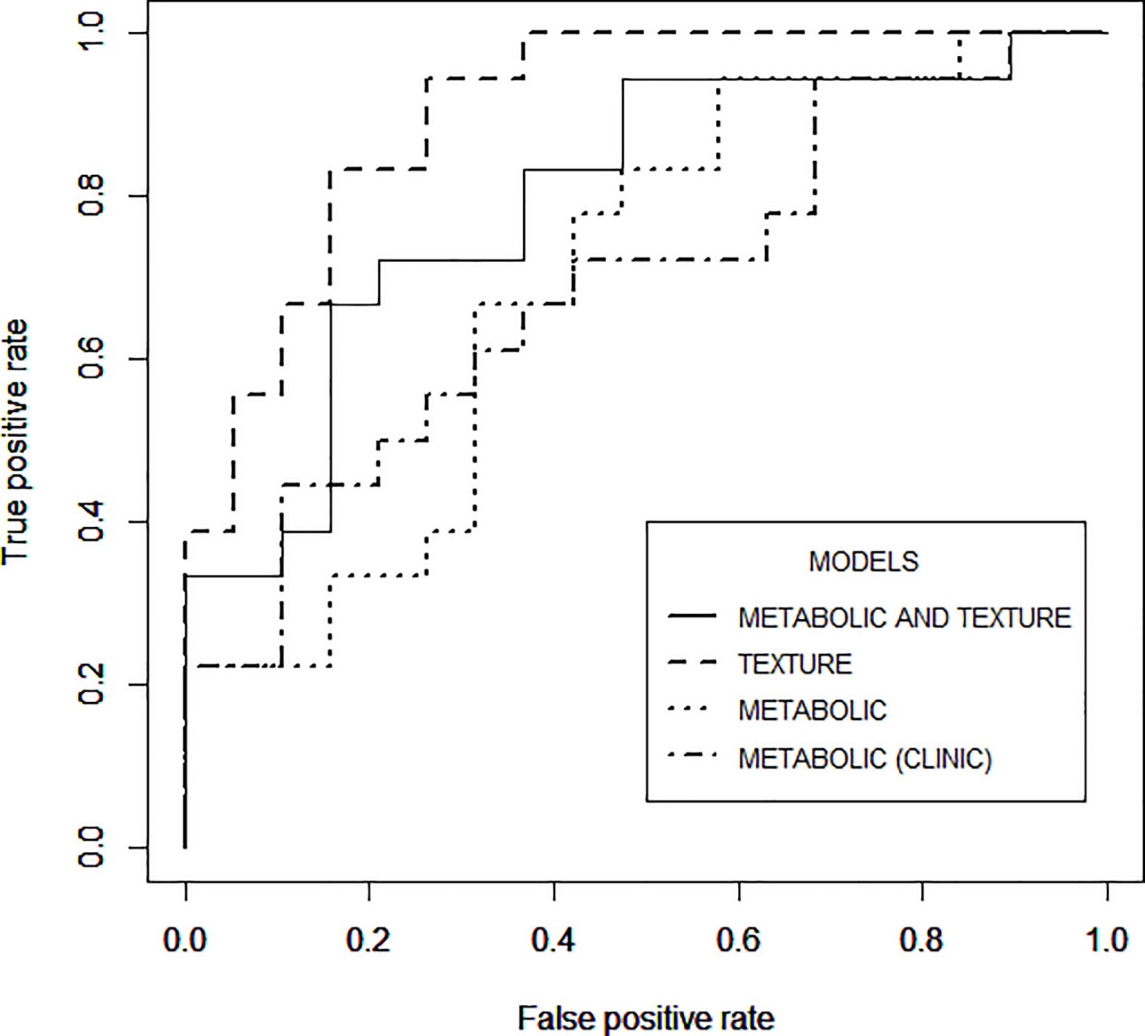
Comparison of the ROC curves using the different sets of features proposed to predict tumor response.

Afterwards, we used multivariate binary logistic regression to study the response predictive ability of the metabolic and quantitative texture features. Multivariate binary logistic regression models were fed with the statistically significant factors that appeared for each set of variables (CLINICAL, ALLMET, TEXTURE, ALLMET-TEXTURE). Their corresponding ROC curves are shown in Fig 4.

When fitting a model with the set of four metabolic features with reported prognosis capacity in previous literature (CLINICAL), only Total Lesion Glycolysis (TLG) resulted statistically significant (*p* = 0.0488). After cross validation, the model obtained an accurate prediction in 50,149±0,293% of the cohort, with an AUC of 0,685±0,010.

When using the whole set of 19 metabolic variables (ALLMET), TLG was no longer significant, while Glycolysis Q1 (*p* = 0,046) remained significant. The results with this model after cross-validation yielded an accuracy of 63,586±0,986% with an AUC of 0,694±0,011.

When entering the 57 automatic texture features (TEXTURE), GLCM Energy at distances of three (*p* = 0,035), five (*p* = 0, 01) and nine voxels (*p* = 0,004) together with IDMN at distance seven voxels (*p* = 0,023) were significant. When cross-validation was performed in this model, an accuracy of 73,051±0,922% with an AUC of 0,815±0,009 were obtained.

When combining metabolic (ALLMET) and texture (TEXTURE) features, Glycolysis Q1 (*p* = 0,041 and GLCM Energy at distances of five (*p* = 0,037) were detected as statistically significant. The results after cross-validation showed an accuracy of 70,154±0,883% with an AUC of 0,768±0, 01. The correlation of radiomic features is presented in S1 Fig.

### Correlation of quantitative PET parameters with biomarkers expression

To study the biological meaning of the PET quantitative parameters that remained significant in the tumor response prediction models above (TLG, Glycolysis Q1, GLCM Energy at distances of three, five and nine voxels and GLCM IDMN at distance of seven voxels), their correlation with VEGFR-2, Ki-67, COX-2, E-cadherin, p-53, BCL-2, c-erb b-2 was studied using Pearson correlation.

VEGFR-2 significantly correlated with GLCM Energy at distance three voxels (r = -0,398, *p* = 0,016) and with IDMN at distance seven voxels (r = -0,374, *p* = 0,025). COX-2 significantly correlated with Glycolysis Q1 (r = -0,366, *p* = 0,024). E- cadherin significantly correlated with GLCM Energy at distance three voxels (r = 0,382, *p* = 0,02). Ki-67 significantly correlated with TLG (r = -0,337, *p* = 0,041) and Glycolysis Q1 (r = -0,366, *p* = 0,026). The degrees of freedom are 35 for all the correlation tests above.

### Correlation of visual and quantitative heterogeneity measurements

To study the relationship between the visual scales proposed and automatic texture, principal components were extracted from TEXTURE database. Ten principal components were obtained with a cumulative variance explained of 90,938%.The absolute value of the contribution of each radiomic feature to each principal component is presented in S2 Fig.

A Spearman correlation matrix was computed including the first five principal components that explained 74,099% of the cumulative variance and both visual scales. The first principal component (explained variance of 33,281%) correlated significantly with visual heterogeneity (r = 0.430, p = 0.048, dof = 35) and visual pattern (r = 0.499, p = 0.02, dof = 35). The rest of the principal components were not significantly correlated with any of the visual scales.

## Discussion

This study shows that tumor heterogeneity in 18F-FDG PET images can discriminate between histopathological responders and non-responders. This information can be of great interest when selecting the best approach for managing colorectal cancer patients as the treatment can be tailored accordingly. The correct identification of non-responders allows their NAT to be intensified. Also, the response prediction could guide optimization of the surgical approach by using less-aggressive alternatives, and even mild postoperative chemotherapy could be prescribed in these cases.

### Predictive capacity of metabolic (SUV related) features

Uptake parameters from PET defining the tumor metabolism (SUV, MTV, and TLG) are the only features used clinically to evaluate the tumor aggressiveness and therapy effectiveness. The prediction capacity of these clinical parameters (CLINICAL) was analyzed to establish the reference level achieved. This reference was later compared to the prediction achieved with the new variables in order to address the relevance of texture parameters. Since results obtained for CLINICAL variables showed poor ability to predict response to NAT, additional parameters related to tumor uptake (ALLMET) were introduced, but only one variable (Glycolysis Q1) remained significant in the multivariate analysis. As shown in Table 2 and Fig 3, only patient information and PET uptake parameters show a poor ability to discriminate between responders and non-responders in this cohort.

### Predictive capacity of texture features

Quantitative heterogeneity features (TEXTURE) seemed to outperform accuracy of metabolic descriptors (AUC of 0,815 and 0,694, respectively). It can be noted that the ability to predict the response is increased as compared with the reference parameters (CLINICAL). When texture is combined with metabolic features (ALLMET-TEXTURE), the AUC reaches 0,768. Given the confidence intervals obtained, the difference of prediction accuracy between using texture alone or combined with metabolic parameters is not significant. These results suggest that the use of texture features may be a promising approach to predict tumor response to NAT.

In the multivariate analysis, several local texture parameters–i.e., study of PET intensity differences in different neighborhoods capturing changes in uptake values of different localities of the tumour—showed significant association with tumor response. The parameters that were significantly associated with response in our model are consistent with others reported previously [27], although there is a high variability in the results obtained with local texture. Soussan *et al*. [21] and Tixier *et al*. [23] reported how GLCM features could predict tumor response to treatment in breast and esophageal cancer. Conversely, Lemarignier *et al*. [42] and Nakajo *et al*. [24] observed no relationship in the same types of cancer. This discrepancy can be due to a GLCM analysis [21,23,24,43] performed at only one-voxel distance, which is a parameter dominated by noise rather than by real intensity differences in this type of images [41,42]. In our work, the use of different distances chosen based on visual differences in intensities, GLCM characteristics showed higher ability to predict tumor response.

No significance was found neither in with global texture metrics (i.e gray-level histogram statistics capturing intensity changes across the whole lesion assuming tumour heterogeneity is well-mixed) nor with regional texture descriptors (i.e. Gray Level Run Length Matrix) in the multivariate analysis. These parameters have been reported to be associated with response and long-term outcome in several types of cancer. Tixier *et al*. [23] and Nakajo *et al* [24] concluded that regional texture descriptors showed better prognostic capacity in esophageal cancer than SUV parameters. Bundschuh *et al*. [26] reported that global texture features could assess response for patients with LARC.

### Biological interpretation of the quantitative texture descriptors

One of the major concerns in radiomics resides in the biological meaning of the parameters used, as the physiological processes underlying texture analysis remain unclear [44]. In this line, we decided to study the correlation between quantitative features significant for response prediction and several key molecules in cancer.

It was shown how the texture features that are able to predict tumor response are significantly correlated with VEGFR and E-Cadherin. VEGFR expression has always been related with angiogenesis and vascular permeability, which are processes characteristic of more aggressive tumors [45]. Thus, this correlation seems to be coherent as new forming blood vessels create local spots and increase heterogeneity of PET images which can be captured by computer-vision quantitative textural features. Moreover, E-cadherin is associated with invasion and metastasis due to the detaching of cancerous cells from the epithelial lining [46]. The association of texture parameters with e-cadherin reinforces the relationship of local heterogeneity in PET with processes in the tumor vessels that may negatively impact tumor prognosis.

Regarding the relationship between because of the glucose metabolic basis of PET imaging, it is not surprising to find that metabolic features correlate with biomarkers related with tumor proliferation (Ki-67) [47] and growth (COX-2) [48]. Nevertheless, it is remarkable that in our series TLG, one of the widely clinically used metabolic parameters, is outperformed by Glycolysis Q1 both in prediction and in the relation with proliferation biomarkers. Glycolysis Q1 refers to the glycolysis calculated on the lower quartile of intensity values. Therefore this might suggest that regions with lower activity concentration–therefore higher Q1 –are related with tumours with lower proliferation rates.

### Visual scores: Easy approach to clinical applicability of the findings

To our knowledge, one of the obstacles to use radiomic features clinically is the complicated relationship with visual appearance of tumors. Thus, we proposed and evaluated a visual classification of heterogeneity to bridge this gap.

Visual heterogeneity and pattern category showed significant association with response to treatment. When both visual scores were introduced, heterogeneity remained significant whereas pattern category did not. Visual scores were then combined with baseline metabolic parameters (CLINICAL). In this case, only heterogeneity scale remained significant in the multivariate model, further supporting the importance of heterogeneity for clinical stratification [19,23,49].

The correlation between the visual scores and quantitative metrics suggests that they are describing similar characteristics. This may aid in the usage of texture features in the clinical procedures as the mathematical texture descriptors can be better understood through their association with the visual score. Besides, this reinforces the necessity of introducing heterogeneity in the medical guidelines for cancer staging as it has clinical significance when evaluating a treatment. Indeed, some PET-derived metrics are already used in the classification and early response assessment of diseases such as lymphomas [50] and trends in PET-imaging feature extraction suggest other types of cancer may also benefit from them [51,52].

We acknowledge several limitations of the study. First, this is a retrospective study with a relatively small sample size without holdout test set available when training the prediction of response. 5-fold cross validation was used to report the findings as a way to reduce biases so our findings suggest significant association between PET parameters and treatment response in LARC but they need to be validated in larger cohorts before claiming any robust prognostic ability. Additionally, the reproducibility of the PET feature findings may depend on the scanner and software. Future guidelines for standardizing procedures remain to be established in the future [23]. Finally, the conclusions can only be applied to patients with LARC, so replication of the study in other pathologies is warranted.

## Conclusion

In this paper, heterogeneity in PET images is shown to be of clinical relevance for the prediction of response to NAT in LARC patients and to have a significant association with key molecular biomarkers in cancer. The main results of this study show how a visual classification of heterogeneity and a further automatic assessment of heterogeneity using texture analysis could become an essential element in research or practical oncology procedures.

Prospective studies are needed to validate the inclusion of these heterogeneity-based metrics as a robust component of the multi-disciplinary approach for the prediction and modelling of response in rectal cancer. This could enable the development of tailored therapies that improve patient´s outcome.

## Supporting information

S1 Fig Correlation matrix of the radiomic features used for training the response prediction model. (TIF)

S2 Fig Absolute value of the weights of the radiomic features describing their contribution in the principal components. (JPG)
