# Supplementary figures and images for: Association of visual and quantitative heterogeneity of 18F-FDG PET images with treatment response in locally advanced rectal cancer: A feasibility study
Source: PLoS One. 2020 Nov 30;15(11):e0242597. doi: 10.1371/journal.pone.0242597 (PMC7704000; doi:10.1371/journal.pone.0242597)

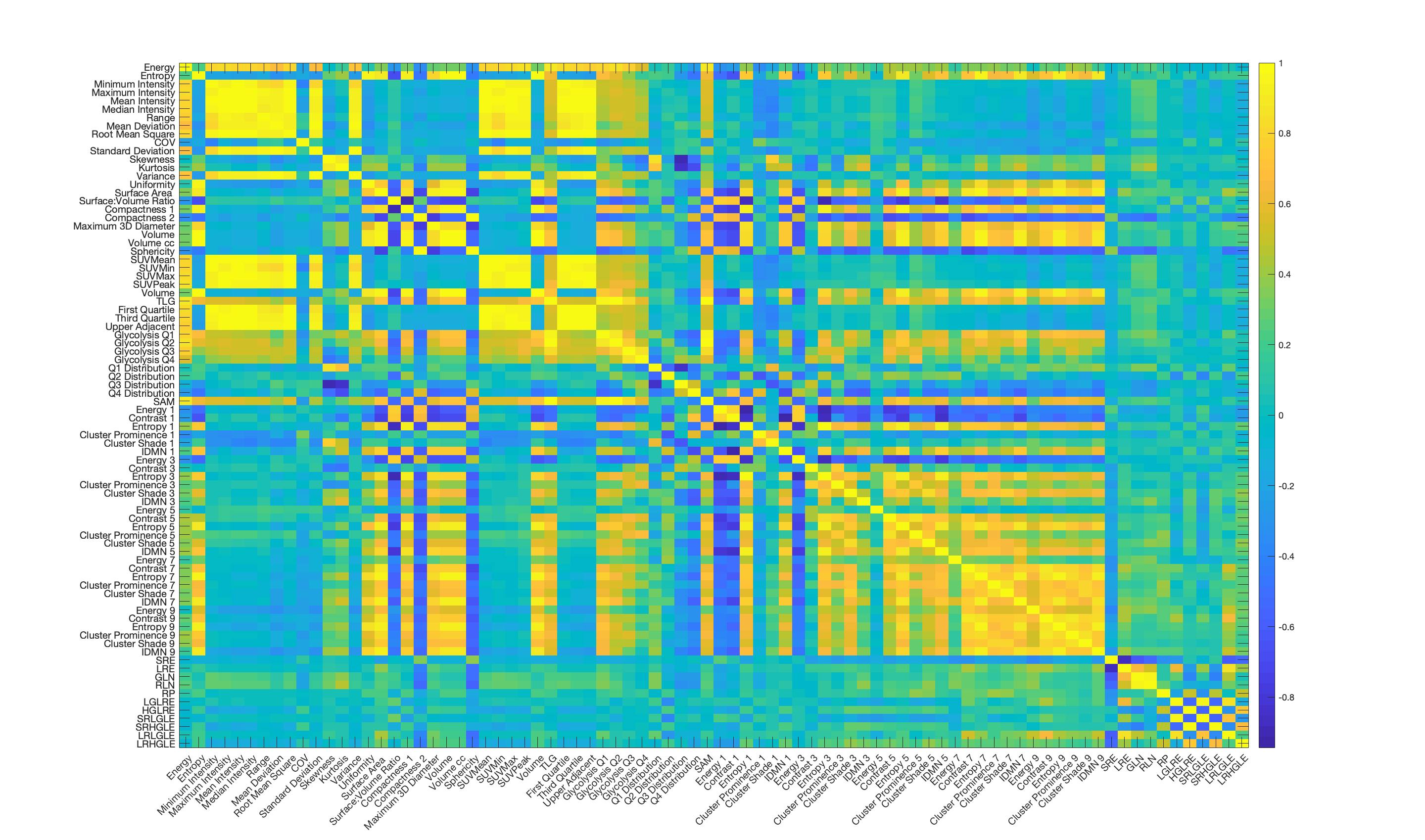

Supplement: S1 Fig — (TIF) [file pone.0242597.s001.tif]

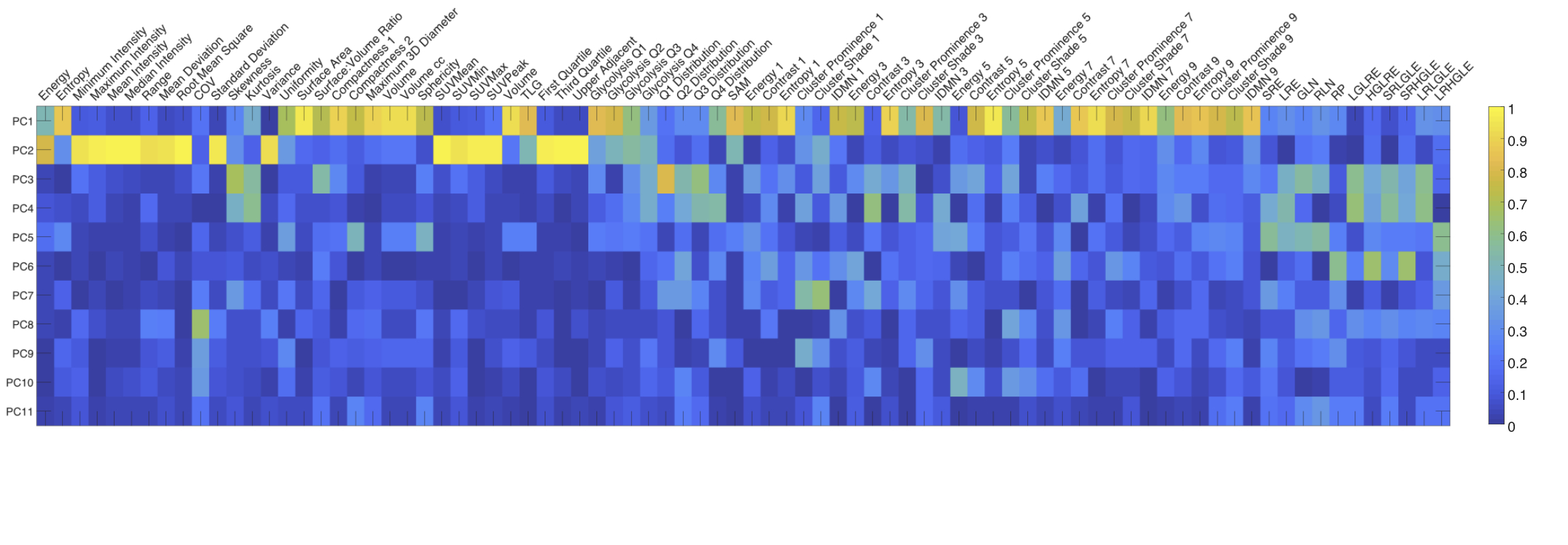

Supplement: S2 Fig — (JPG) [file pone.0242597.s002.jpg]
